# Changes in eating habits following total and frontolateral laryngectomy

**DOI:** 10.1590/S1516-31802004000500003

**Published:** 2004-09-01

**Authors:** Jackeline Pillon, Maria Inês Rebelo Gonçalves, Noemi Grigoletto De Biase

**Keywords:** Food habits, Deglutition disorder, Laryngectomy, Deglutition, Head and neck neoplasms, Hábitos alimentares, Transtornos de deglutição, Laringectomia, Deglutição, Neoplasias de cabeça e pescoço

## Abstract

**CONTEXT:**

Swallowing is a continuous dynamic process, characterized by complex stages, that involves structures of the oral cavity, pharynx, larynx and esophagus. It can be divided into three phases: oral, pharyngeal and esophageal. Dysphagia is characterized by difficulty with, or the inability to swallow food of normal consistencies.

**OBJECTIVE:**

To investigate the presence of swallowing difficulties and modifications made to the consistency of the food consumed in cases of total and partial laryngectomy, with or without subsequent radiotherapy, among patients who had not been diagnosed as having dysphagia.

**TYPE OF STUDY:**

Descriptive study.

**SETTING:**

Voice Clinic of São Paulo Hospital, Universidade Federal de São Paulo/Escola Paulista de Medicina, São Paulo, Brazil.

**METHOD:**

36 laryngectomy patients: 25 total and 11 frontolateral cases, were studied. A survey consisting of a 23-item questionnaire was applied by a single professional.

**RESULTS:**

Among those interviewed, 44% reported having modified the consistency of the food consumed (56% of the total and 20% of the partial frontolateral laryngectomy cases). It was not possible to investigate the influence of radiotherapy on the groups in this study, because the partial frontolateral laryngectomy cases were not exposed to radiotherapy. There was a higher incidence of complaints of swallowing difficulties in total laryngectomy cases (p < 0.027) than in partial frontolateral cases. However, there was no relationship between the surgery and weight loss. We also noted the patients' other problems regarding the eating process, as well as the compensation that they made for such problems.

**DISCUSSION:**

Research has shown an association between laryngectomy and swallowing difficulties, although there have been no reports of associated changes in eating habits among laryngectomized patients.

**CONCLUSIONS:**

This study showed that difficulty in swallowing is not rare in total and frontolateral laryngectomy cases. Such patients, even those who did not complain of dysphagia, also had minor difficulties while eating, and had to make some adaptations to their meals.

## RESUMO

**CONTEXTO:**

A deglutição é um processo dinâmico e contínuo, caracterizado por etapas complexas, que envolve estruturas da cavidade oral, faringe, laringe e esôfago. Pode ser dividida em fases: oral, faríngea e esofágica. A disfagia é caracterizada por dificuldades ou inabilidade em deglutir alimentos em consistências normais.

**OBJETIVO:**

Investigar a presença de dificuldades de deglutição e mudanças nas consistências dos alimentos em pacientes submetidos a laringectomia total e laringectomia parcial vertical frontolateral, seguidos ou não de radioterapia e sem diagnóstico de disfagia.

**TIPO DE ESTUDO:**

Descritivo.

**LOCAL:**

Ambulatório de Voz do Hospital São Paulo, Universidade Federal de São Paulo — Escola Paulista de Medicina, São Paulo, Brazil.

**MÉTODO:**

36 pacientes laringectomizados: 25 submetidos a laringectomia total e 11 a laringectomia parcial frontolateral foram incluídos no estudo.

Questionário contendo 23 itens, foi aplicado por uma única profissional.

**RESULTADOS:**

Dentre os entrevistados, 44% relataram modificações na consistência dos alimentos durante as refeições; dentre estes 56% eram laringectomizados totais e 20%, laringectomizados parciais frontolaterais. Não foi possível investigar a influência da radioterapia nos grupos deste estudo, pois os pacientes laringectomizados parciais frontolaterais não foram expostos à radioterapia. Houve alta incidência de queixas de dificuldades de deglutição entre os pacientes laringectomizados totais (p < 0,027). Entretanto, não houve associação entre cirurgia realizada e perda de peso. Observamos outras queixas relatadas pelos pacientes durante a alimentação, como problemas e compensações realizadas pelos pacientes.

**DISCUSSÃO:**

Pesquisas mostraram associação entre laringectomia e dificuldades de deglutição, porém não foram encontrados estudos associando mudanças de hábitos alimentares em pacientes laringectomizados.

**CONCLUSÃO:**

Este estudo mostra que a dificuldade na deglutição não é rara nos pacientes submetidos a laringectomia frontolateral e total. Os pacientes, mesmo aqueles que não apresentaram queixas de disfagia, também tiveram pequenas dificuldades durante a alimentação, fazendo algumas adaptações necessárias.

## INTRODUCTION

Swallowing may seem to be a simple process, yet it involves complex stages. It is a dynamic process of short duration, and is divided into four parts: the preparatory phase, the oral phase, the pharyngeal phase, and the esophageal phase.^1^ The swallowing process begins with the voluntary movements of the oral phase, and it continues in an involuntary manner, in the pharyngeal and esophageal phases.

Difficulty in swallowing is called dysphagia, and it involves an inability to manage the entire process of eating food of normal consistency.^2^ Dysphagia has several causes: dysphagia of mechanical origin is generally due to trauma, or is a consequence of the resection of head and neck tumors.^3^

Laryngectomy patients do not necessarily complain of difficulty in swallowing. However, the small deviations resulting from the surgery may require modifications to the consistency of their food, in order to make it easier to eat. Thus, such patients usually prefer soft or liquid diets in order to facilitate the eating process, and tend to avoid certain types of food, particularly solids, that are more difficult to swallow, especially when there are mechanical dysfunctions associated. Such dietary changes may impair the patient's quality of life.

Several types of surgery have been described in the literature for the treatment of laryngeal cancer: partial, subtotal and total. Partial frontolateral laryngectomy is described as the resection of the carina of the thyroid cartilage, plus the subperichondral excision of a vocal fold, with or without an arytenoidectomy. In this surgery, the resection involves the glottic level of the larynx. Since the vocal folds also carry out sphincteric action during the swallowing process, the resection excludes their participation in the adduction, thereby causing an alteration in the swallowing process, as well as in the formation of sounds.^4^

The treatment following surgery for the removal of cancer may often be radiotherapy. This may cause subsequent reactions such as necrosis of the tissue, edema of the larynx and fibrosis or hypertonia around the pharyngoesophageal and/or esophageal sphincter. The passage of the food products to the esophagus may thus be hindered because of the reduction in extent of the peristaltic movements.^3^

Along with the radiotherapy, it may be advisable to use a nasogastric tube, or even to modify the consistency of the food consumed. Weight loss occurs if the alterations persist or if there are acute complications in the irradiated tissue, which would generally consist of necrosis of the tissue.^3^

Partial frontolateral laryngectomy cases may present insufficient elevation of the larynx. Insufficiency or absence of laryngeal elevation would result in a lack of protection under the base of the tongue during the pharyngeal phase of swallowing, and the food could be inhaled into the lungs.^5,6^ In total laryngectomy cases, the whole larynx is removed, and as a result, the respiratory and digestive passageways are completely separated. With the removal of the larynx, this separation becomes permanent when the proximal stump of the trachea is closed and the anterior region of the neck is opened. This procedure, which allows for breathing, is called a tracheostomy.^4^ In such cases, speech function is most jeopardized, while dysphagia only occurs in a few of these cases.^7^

Dysphagia in total laryngectomy cases may be related to tumor recurrence,^7,8^ presence of a second primary tumor in the esophagus, rigidity of the pharyngeal musculature due to radiotherapy, formation of a pseudoepiglottis,^5^ or regurgitation of food and uncoordinated contraction of the pharyngeal constrictor muscles.^8^

Awareness of the effects of changes in swallowing due to laryngeal surgery enables the professional to perform the pre-surgical contacts and post-surgical rehabilitation better.

Thus, the objective of this study was to investigate the swallowing difficulties and the modifications made to the consistency of food consumed in cases of total and partial frontolateral laryngectomy, with or without subsequent radiotherapy, among patients who had not been diagnosed as having dysphagia.

## METHODS

*Design:* Descriptive study.

*Setting:* This study was performed at the Voice Clinic of the São Paulo Hospital, Universidade Federal of São Paulo, which is a public institution.

*Participants:* 36 patients who did not have dysphagia, of whom 29 (80%) were male and 7 (20%) were female. They were interviewed over a period of one month. All the patients were attending the Voice Clinic of the São Paulo Hospital and freely agreed to participate in the interview. No patient was excluded from the survey. The patients were invited to come, and were informed about the study and the need for their compliance for the duration of the commitment. All the participants in this study had had surgery to remove a tumor. There were 25 total laryngectomy cases (70%) and 11 partial frontolateral laryngectomy cases (30%). The length of time since their operations was a minimum of one month and maximum of six years. Two years was the average length of time. All of the patients who received radiotherapy were cases of total laryngectomy. With regard to complaints of frequent eructation and heartburn (gastroesophageal reflux) in our study, no medical evaluation was done to verify the presence of gastroesophageal reflux, since such complaints were considered to be indications of these. Patients who had had laryngeal surgery in relation to the resection of other structures, such as glossectomy, were excluded from the study.

*Procedures and main measurements:* Initially, a specific questionnaire was developed containing 23 questions. All patients were interviewed by the same professional (a speech, hearing and language pathologist) who gave them instructions and answered their questions. The patients were interviewed through the use of a specific semi-structured questionnaire requesting demographic information (age, sex and profession). They were asked about their type of surgery and radiation therapy, and for information on dysphagia by means of indirect questions regarding their problems during eating, such as difficulties while ingesting food of different consistencies, coughing, weight loss, breathing discomfort, choking, suffocating, pharyngeal globus, swallowing noises, regurgitation, sensation of food stuck in the throat, frequent eructation and heartburn. They were also asked about the compensation they made, such as modifying the consistency of the food consumed, reducing the quantity ingested, multiple swallowing, and head maneuvers.

*Statistical methods:* The Fisher statistical test was applied to the specific variables mentioned. This test reveals any differences between the groups. Results with p < 0.05 were considered to be significant and are marked with an asterisk (*).

## RESULTS

Table 1 show the frequencies of complaints in the groups of total and partial frontolateral laryngectomy patients. In these groups, 13 of the patients (36%) had difficulties while eating: 12 (48%) were total laryngectomy cases and one (9%) was a partial frontolateral laryngectomy case. The comparison between the groups showed a statistically significant relationship (p < 0.027).

**Table 1:** Comparison between surgery types and complaints (n = 36)

Complaints	Surgery							
	Total laryngectomy (n = 25)				Partial frontolateral laryngectomy (n = 11)			
	Yes		No		Yes		No	
	N	%	N	%	N	%	N	%
Swallowing difficulties	12*	48	13	52	1*	9	10	91
Modifications to food consistency	14	56	11	44	2	20	9	80
Weight loss	15	60	10	40	3	27	8	73
Swallowing difficulties after radiation therapy	9	69	4	31	0	0	0	0

* *p < 0.027.*

There was no statistically significant difference (p = 0.067) between the two groups interviewed in relation to modifications to the consistency of the food consumed. We noted that 14 (56%) of the total laryngectomy patients mentioned making such modifications, while two patients (20%) with partial frontolateral laryngectomy made such modifications.

Fifteen of the total laryngectomy patients (60%) reported weight loss after the surgery. Of the patients submitted to partial frontolateral laryngectomy, only three (27.3%) presented this complaint. None of the patients had permanent weight loss. There was no statistically significant difference (p = 0.073) in relation to complaints of weight loss in these groups.

Only the total laryngectomy cases received radiotherapy and, of these, nine (69%) had difficulties in swallowing food. Therefore it was not possible to investigate the influence of radiotherapy on the two groups.

Other eating difficulties were also reported by the patients (Table 2). Among these problems reported by total laryngectomy patients, the most significant related to symptoms of gastroesophageal reflux (p < 0.016). Also reported were coughing, choking, sensation of food stuck in the throat, suffocation, swallowing noises, pharyngeal globus, breathing discomfort and regurgitation. The patients described the compensation they made during eating in terms of head maneuvers, decreased daily food intake and swallowing maneuvers.

**Table 2:** Comparison between surgery types and problems and compensation (n = 36)

	Total Laryngectomy (n = 25)	Partial frontolateral laryngectomy (n = 11)	p-value (Indifference test)
**Problems**			
Reflux	13	1	0.016*
Coughing	7	3	0.647
Choking	7	1	0.210
Sensation of food stuck	6	1	0.291
Suffocation	4	0	0.215
Swallowing noises	4	0	0.215
Regurgitation	2	0	0.476
Pharyngeal globus	2	1	0.678
Breathing discomfort	2	0	0.476
**Compensation**			
Head maneuvers	22	10	0.644
Decreased food intake	14	3	0.109
Multiple swallowing	6	0	0.091

* *p < 0.016.*

## DISCUSSION

Several studies have evaluated the quality of life among patients who have had cancer removed from the head and neck.^9,10^ In the present study we investigated the modifications to eating habits among patients submitted to total and partial frontolateral laryngectomy.

We defined dysphagia according to Ward (2002), who considered dysphagia to be any inability to administer food of normal consistency. This author showed that laryngectomy patients who suffered from eating disabilities for long periods of time were negatively affected, resulting in anguish and subsequent alteration of the patients’ quality of life.^2^

For the two types of surgery considered, which have different reconstruction methods, our study showed common patterns and solutions for facilitating swallowing. The most frequent were modifications to the consistency of the food and head maneuvers. Multiple swallowing was only reported by patients submitted to total laryngectomy, representing 25% of this group.

Eating difficulties among laryngectomy patients may be related to the radiotherapy that they were submitted to after surgery. Radiotherapy causes an inflammatory processes in the area and can alter pharyngeal sensitivity and motricity, thus interfering with swallowing.^3^

In the present study, only the total laryngectomy patients were exposed to radiotherapy. Of the patients that had radiotherapy, we observed that nine (69%) reported difficulty in swallowing. Such findings suggest that radio-therapy is an important factor in the occurrence of eating difficulties, particularly in relation to the ingestion of solids. In this study, there was no detailed investigation regarding how radiotherapy may have influenced dysphagia. Further studies would be necessary, as shown in the literature.^6,7,11^ However, we may consider that radiotherapy did have an influence on the total laryngectomy patients of the present study because, according to the literature, the consequences of radiotherapy can be rigidity of the pharyngeal musculature,^5^ regurgitation, uncoordinated contraction of the pharyngeal constrictor muscles^8^ and fistulas.^12^

When we compared the eating difficulties between the two groups of laryngectomy patients, we observed that in total laryngectomy cases, 48% reported difficulties while eating. Such difficulties were related to solid food in 60% of the cases.

Studies among normal individuals have shown that increased food viscosity requires greater action by the pharyngeal muscles.^13^ Thus, we can conclude that radiotherapy had a direct influence in hindering the swallowing of solids, due to the harming of the pharyngeal muscles.

In the partial frontolateral laryngectomy cases, swallowing impairment may have been present because of the types of surgical reconstruction done. There was a statistically significant difference (p < 0.027) in relation to the type of surgery. The incidence of complaints of swallowing difficulties was greater among patients submitted to total laryngectomy. In this type of surgery, the respiratory and upper digestive passageways become anatomically separated when the proximal stump of the trachea is closed, after removal of the larynx.^4^ Thus, the passageways end up being used for food alone, and the air goes by a separate route.

The eating difficulties experienced by total laryngectomy patients may be related to tumor recurrence,^7,8^ presence of a second primary tumor in the esophagus, rigidity of the pharyngeal musculature due to radiotherapy, formation of a pseudoepiglottis,^5^ or regurgitation of food and uncoordinated contraction of the pharyngeal constrictor muscles.^8^ These variables were not investigated in this study.

Eating difficulties experienced by partial frontolateral laryngectomy patients may occur because of inadequate laryngeal elevation, which would hinder the protection of the lower air passageways below the base of the tongue, during the pharyngeal phase of swallowing.^5^ This difficulty can be avoided by carrying out swallowing maneuvers, but the best solution would be the adoption of a better consistency of food for such patients.

Balfe et al.^7^ evaluated 45 patients submitted to total laryngectomy and reported that dysphagia was diagnosed in seven (16%) of them. Our data differ from theirs, because approximately half of the total laryngectomy group experienced some degree of difficulty in eating. However, Pauloski et al.^14^ noted among 352 patients with various lesions caused by laryngeal cancer that 59% complained of swallowing difficulties.

Weight loss soon after surgery is often due to the period of adaptation experienced by the patient, because of the use of a feeding tube until rehabilitation has been completed and the patient returns to oral ingestion, in accordance with the type of laryngectomy performed. During this period, the patient may also be given radiotherapy, which can cause swelling of the affected area, pain during swallowing, or xerostomia, among other complications.^3^ When this period has passed, the patient is able to eat better and maintain his weight.^15^

In our study, it was noted that patients only reported weight loss just after the surgery. This suggests that, even with eating difficul-ties, the patients were seeking ways of adapting their eating habits because, at the time when they were interviewed, these patients were having vocal rehabilitation therapy, and so there were no complaints of weight loss.

It has been shown that dysphagia among laryngectomy patients is reduced when the nasoenteral feeding tube is removed, although some difficulty in swallowing remained.^15^ Other studies have shown that total laryngectomy cases present late-stage complications, most frequently involving dysphagia.^7,16^ A single study has reported that 63% of the patients with head and neck cancer had decreased food intake for 30 days after the surgery.^9^ In our study, we also observed other complaints related to eating, as reported by the patients. Among the problems reported, reflux was the most frequent one among total laryngectomy patients. In this study, no detailed investigations were made for the diagnosis of reflux. The other problems reported were coughing, sporadic gagging, sensation of food stuck in the throat, suffocation, swallowing noises, pharyngeal globus and breathing discomfort. Among the compensation for the problems that patients reported, head maneuvers were the most frequently reported, followed by decreased food intake and multiple swallowing.

Since the impact of the removal of the larynx is enormous and not restricted to just one alteration, it is to be expected that patients might have several types of complaints, problems and compensation. There is also difficulty in communication, which is probably the function that is most jeopardized. Because of the communication difficulties, the importance of eating problems may be minimized in the eyes of patients and the attending team, even to the point of neglecting such alterations during consultations.

As part of the multidisciplinary team, the speech-hearing-language pathologist is able to investigate the question of dysphagia, as well as the alterations and adaptations of eating patterns. The speech-hearing-language pathologist can provide the opportunity for the patient to talk about the different aspects of the situation, so as to improve the patient's quality of life in as effective a way as possible, within the limitations caused by the surgery.

In our study, further investigation was not done regarding the etiology of the problems reported by the patients. Further studies are needed, by means of videofluoroscopic examination, as has been done in studies in other countries.^6,7,11,17,18^ It is important to point out that the questions raised in this study were not frequent patient complaints, because these patients were attending therapy sessions for the rehabilitation of the esophageal voice. It was only when questioned about possible eating difficulties that they began to think about the matter and ask questions about the difficulties they were experiencing.

With this study, it becomes evident that health professionals who attend to patients who have had laryngeal tumors removed ought to always be alert to any difficulties or complaints, and should proceed with more precise examination of patients’ difficulties and the etiologies of such difficulties.

## CONCLUSION

This study has shown that dysphagia is not rare in cases of total and partial frontolateral laryngectomy.

Modifications to the consistency of the food consumed, use of head maneuvers and decreased food intake were the methods adopted for facilitating swallowing.

Considering the two types of surgery studied, patients who underwent total laryngectomy were the ones who reported the most difficulty in swallowing.
